# *N*-(5-Nitro­pyridin-2-yl)-5*H*-dibenzo[*d*,*f*][1,3]diazepine-6-carboxamide

**DOI:** 10.1107/S1600536811018629

**Published:** 2011-05-25

**Authors:** Tomasz Seidler, Marlena Gryl, Bartosz Trzewik, Katarzyna Stadnicka

**Affiliations:** a Faculty of Chemistry, Jagiellonian University, R. Ingardena 3, 30-060 Kraków, Poland

## Abstract

The title compound, C_19_H_13_N_5_O_3_, can be obtained from the corresponding α-amido-α-amino­nitrone in a reaction with biphenyl-2,2′-diamine. The amido–amidine core has distinctive geometrical parameters including: an outstandingly long C*sp* ^2^—C*sp* ^2^ single bond of 1.5276 (13) Å and an amidine N—C—N angle of 130.55 (9)°. Intra­molecular N—H⋯O, N—H⋯N and C—H⋯O hydrogen bonds occur. In the crystal, mol­ecules form layers parallel to (001) *via* weak inter­molecular C—H⋯N inter­actions. The layers are linked *via* N—H⋯O hydrogen bonds and π–π inter­actions along [001] [benzene–pyridine centroid–centroid distance = 3.672 (2) Å].

## Related literature

For the synthesis of the title compound, see: Trzewik *et al.* (2008 ▶). For the reaction mechanism, see: Trzewik *et al.* (2010 ▶). For similar structures, see: Zaleska *et al.* (2007 ▶); Hodorowicz *et al.* (2007 ▶). For hydrogen bond graph-set analysis, see: Bernstein *et al.* (1995 ▶). 
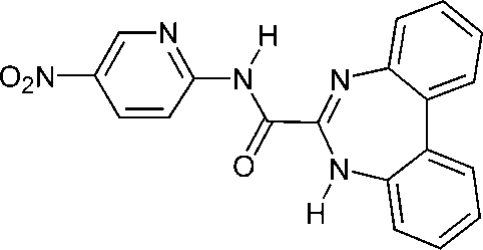


## Experimental

### 

#### Crystal data

C_19_H_13_N_5_O_3_
*M* *_r_* = 359.34
Monoclinic, 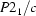

*a* = 12.9702 (2) Å
*b* = 9.2104 (1) Å
*c* = 13.4145 (2) Å
β = 100.692 (1)°
*V* = 1574.68 (3) Å^3^
*Z* = 4
Mo *K*α radiation
μ = 0.11 mm^−1^
*T* = 110 K
0.30 × 0.20 × 0.15 mm

#### Data collection

Oxford Diffraction SuperNova Dual Cu at zero Atlas diffractometer
Absorption correction: multi-scan (*CrysAlis PRO*; Oxford Diffraction, 2009 ▶) *T*_min_ = 0.969, *T*_max_ = 0.984
127481 measured reflections
4577 independent reflections
3784 reflections with *I* > 2σ(*I*)
*R*_int_ = 0.052

#### Refinement

*R*[*F* ^2^ > 2σ(*F* ^2^)] = 0.036
*wR*(*F* ^2^) = 0.110
*S* = 1.06
4577 reflections
250 parameters
2 restraints
H atoms treated by a mixture of independent and constrained refinement
Δρ_max_ = 0.39 e Å^−3^
Δρ_min_ = −0.21 e Å^−3^

### 

Data collection: *CrysAlis PRO* (Oxford Diffraction, 2009 ▶); cell refinement: *CrysAlis PRO*; data reduction: *CrysAlis PRO*; program(s) used to solve structure: *SIR92* (Altomare *et al.*, 1994 ▶); program(s) used to refine structure: *SHELXL97* (Sheldrick, 2008 ▶); molecular graphics: *Mercury* (Macrae *et al.*, 2006 ▶) and *ORTEP-3* (Farrugia, 1997 ▶); software used to prepare material for publication: *SHELXL97*, *PARST* (Nardelli, 1995 ▶) and *WinGX* (Farrugia, 1999 ▶).

## Supplementary Material

Crystal structure: contains datablocks global, I. DOI: 10.1107/S1600536811018629/vm2095sup1.cif

Structure factors: contains datablocks I. DOI: 10.1107/S1600536811018629/vm2095Isup2.hkl

Supplementary material file. DOI: 10.1107/S1600536811018629/vm2095Isup3.cml

Additional supplementary materials:  crystallographic information; 3D view; checkCIF report

## Figures and Tables

**Table 1:** Hydrogen-bond geometry (Å, °)

*D*—H⋯*A*	*D*—H	H⋯*A*	*D*⋯*A*	*D*—H⋯*A*
N2—H2⋯O4	0.89 (1)	2.24 (1)	2.7041 (11)	112 (1)
N2—H2⋯O4^i^	0.89 (1)	2.26 (1)	3.0725 (11)	152 (1)
N5—H5⋯N3	0.88 (1)	2.11 (1)	2.6191 (11)	116 (1)
C55—H55⋯O4	0.95	2.33	2.9266 (12)	120
C32—H32⋯N51^ii^	0.95	2.47	3.3000 (13)	146

Symmetry codes: (i) 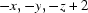; (ii) 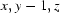.

**Fig. 1.**
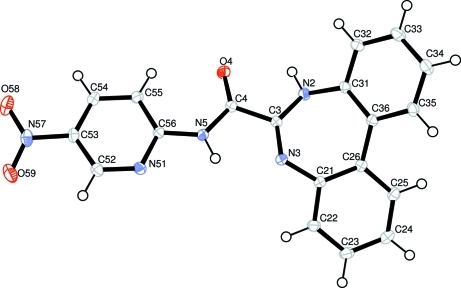
Asymmetric unit of the title compound showing the atom displacement ellipsoids drawn at the 50% probability level. H atoms are shown as spheres of arbitrary radii.

**Fig. 2.**
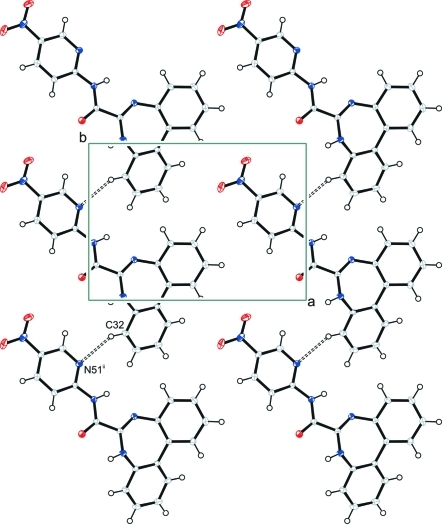
The hydrogen bond scheme in the layer parallel to (001) cut for z in the range 0.75 to 1.00 (symmetry code: (ii) x, y-1, z).

**Fig. 3.**
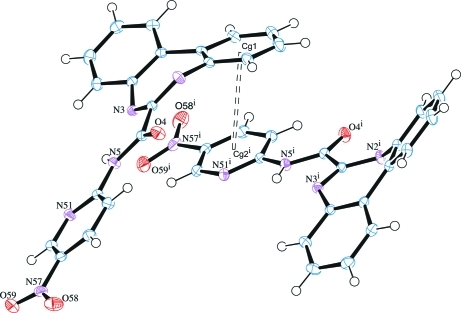
A diagram of π—π interactions between Cg1 (C31–C36) and Cg2i (N51–C56) (symmetry code: (i) -x, y + 1/2, -z + 3/2).

**Fig. 4.**
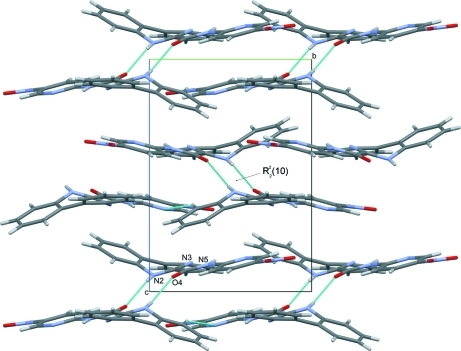
View of the packing along [001] showing hydrogen bonds between the layers and the ring motif with descriptor R22(10).

## supplementary crystallographic information

### Comment

The current report is a continuation of an earlier joint theoretical and X-ray study upon the versatile reactivity of α-amido-α-aminonitrones, having several reactivity centers of different types and yielding various products in reactions with electrophilic and nucleophilic reagents (Trzewik *et al.*, 2008). Among them 5*H*-dibenzo[*d*,*f*][1,3]diazepines, the synthesis and structures of which were described elsewhere (Trzewik *et al.*, 2008, 2010), are unique from the viewpoint of their geometrical features.

The overall shape of the title molecule is shown in Figure 1. The two benzene rings within the diazepine moiety are twisted by torsion angle C25—C26—C36—C35 = -28.63 (13)*^o^*. The r.m.s. deviation for the best plane through atoms C21-C26 is significantly greater than that for C31-C36 (0.0166 and 0.0040 Å, respectively) due to steric hindrance between H25 and H35 (H25···H35 distance 2.12 Å).

The puckering parameters of the seven-membered ring (atoms in C3, N2, C31, C36, C26, C21, N3 sequence): q_2_ = 0.5324 (9), q_3_ = 0.0832 (9), QT = 0.5389 (9), φ_2_ = 87.6 (1), φ_3_ = 12.4 (7), θ_2_ = 81.1 (1)°, indicate a twisted-boat conformation with a pseudo-twofold axis (C_2_) through the C3 atom and the centre of C36—C26 bond with the deviation of 0.0369 (4) Å, whereas a pseudo-mirror plane (Cs) through N2 atom and centre of C21— C26 is described by the deviation of 0.0491 (5) Å (*PARST*: Nardelli, 1995).

The rest of the molecule is almost perfectly planar (r.m.s. deviation of fitted atoms equals 0.0181 Å). The fragment of the molecule, relevant from both crystallographic and chemical perspectives, is the amido-amidine core [—N5(—H5)—C4(=O4)—C3(=N3—)—N2(—H2)—]. Within the core distinctive geometrical features of the molecule can be seen: a long C3(*sp*^2^)—C4(*sp*^2^) bond of 1.528 (1) Å and N2—C3—N3 angle of 130.55 (9)°. We expect that the planarity of the core moiety possibly results from intramolecular interactions: N5—H5···N3, N2—H2···O4 and C55—H55···O4 (Table 1). In order to verify the existence of such interactions the analysis of topological properties of electron density distribution is in progress and will be published elsewhere.

The packing of the molecules is organized into layers parallel to (001). Within the layer the molecules are joint by hydrogen bonds of C–H···N type and weak interactions (Figure 2, Table 1). The layers are joined together by π—π interactions with *Cg*1 (C31–C36)—*Cg*2 (N51-C56) [-*x*, *y* + 1/2, -*z* + 3/2] = 3.672 Å (Figure 3); and hydrogen bonds of N—H···O type. The N—H···O hydrogen bond together with its centrosymmetric counterpart form a ring motif with descriptor *R*_2_^2^(10) according to graph-set theory (Bernstein *et al.*, 1995). The ring motif is marked in Figure 4.

### Experimental

The title compound was synthesized using the procedure already described in literature (Trzewik *et al., 2*008). Single crystals suitable for X-ray diffraction were grown by slow evaporation from the mixture of methanol and acetonitrile (1:2) solution at ambient conditions.

### Refinement

All hydrogen atoms of N—H groups were found in difference Fourier maps and refined in a riding model assuming N—H = 0.88 (2) Å and *U*_iso_ = 1.2*U*_eq_ of the parent atom. Aromatic hydrogen atoms were found in difference Fourier maps and refined from geometrical positions assuming C—H = 0.95 Å and using riding model with *U*_iso_ = 1.2*U*_eq_.

### Crystal data

**Table:**

C_19_H_13_N_5_O_3_	*F*(000) = 744
*M**_r_* = 359.34	*D*_x_ = 1.516 Mg m^−^^3^
Monoclinic, *P*2_1_/*c*	Melting point = 477–478 K
Hall symbol: -P 2ybc	Mo *K*α radiation, λ = 0.71073 Å
*a* = 12.9702 (2) Å	Cell parameters from 34515 reflections
*b* = 9.2104 (1) Å	θ = 3.0–44.5°
*c* = 13.4145 (2) Å	µ = 0.11 mm^−^^1^
β = 100.692 (1)°	*T* = 110 K
*V* = 1574.68 (3) Å^3^	Block, orange
*Z* = 4	0.30 × 0.20 × 0.15 mm

### Data collection

**Table:**

Oxford Diffraction SuperNova Dual Cu at zero Atlas diffractometer	4577 independent reflections
Radiation source: Oxford Diffraction SuperNova (Mo) X-ray Source	3784 reflections with *I* > 2σ(*I*)
mirror	*R*_int_ = 0.052
Detector resolution: 10.3756 pixels mm^-1^	θ_max_ = 30.0°, θ_min_ = 3.0°
ω scans	*h* = −18→17
Absorption correction: multi-scan (*CrysAlis PRO*; Oxford Diffraction, 2009)	*k* = 0→12
*T*_min_ = 0.969, *T*_max_ = 0.984	*l* = 0→18
127481 measured reflections	

### Refinement

**Table:**

Refinement on *F*^2^	Secondary atom site location: difference Fourier map
Least-squares matrix: full	Hydrogen site location: difference Fourier map
*R*[*F*^2^ > 2σ(*F*^2^)] = 0.036	H atoms treated by a mixture of independent and constrained refinement
*wR*(*F*^2^) = 0.110	*w* = 1/[σ^2^(*F*_o_^2^) + (0.0729*P*)^2^ + 0.1623*P*] where *P* = (*F*_o_^2^ + 2*F*_c_^2^)/3
*S* = 1.06	(Δ/σ)_max_ < 0.001
4577 reflections	Δρ_max_ = 0.39 e Å^−^^3^
250 parameters	Δρ_min_ = −0.21 e Å^−^^3^
2 restraints	Extinction correction: *SHELXL97* (Sheldrick, 2008)
0 constraints	Extinction coefficient: 0
Primary atom site location: structure-invariant direct methods	

### Special details

**Table:**

Experimental. CrysAlisPro, Oxford Diffraction Ltd., Version 1.171.33.66. Empirical absorption correction using spherical harmonics, implemented in SCALE3 ABSPACK scaling algorithm (Oxford Diffraction, 2009).
Geometry. All e.s.d.'s (except the e.s.d. in the dihedral angle between two l.s. planes) are estimated using the full covariance matrix. The cell e.s.d.'s are taken into account individually in the estimation of e.s.d.'s in distances, angles and torsion angles; correlations between e.s.d.'s in cell parameters are only used when they are defined by crystal symmetry. An approximate (isotropic) treatment of cell e.s.d.'s is used for estimating e.s.d.'s involving l.s. planes.
Refinement. Refinement of *F*^2^ against all reflections. The weighted *R*-factor *wR* and goodness of fit *S* are based on *F*^2^, conventional *R*-factors *R* are based on *F*, with *F* set to zero for negative *F*^2^. The threshold expression of *F*^2^ > 2σ(*F*^2^) is used only for calculating *R*-factors(gt) *etc*. and is not relevant to the choice of reflections for refinement. *R*-factors based on *F*^2^ are statistically about twice as large as those based on *F*, and *R*- factors based on all data will be even larger.

### Fractional atomic coordinates and isotropic or equivalent isotropic displacement parameters (Å^2^)

**Table:**

	*x*	*y*	*z*	*U*_iso_*/*U*_eq_	
N2	0.15616 (7)	0.02467 (9)	0.92909 (6)	0.01621 (17)	
H2	0.1014 (9)	−0.0115 (14)	0.9524 (9)	0.019*	
C3	0.14265 (7)	0.16642 (10)	0.90162 (7)	0.01300 (18)	
N3	0.20711 (6)	0.25840 (9)	0.87513 (6)	0.01359 (16)	
C4	0.03385 (7)	0.22423 (10)	0.90872 (7)	0.01420 (18)	
O4	−0.03207 (5)	0.14658 (8)	0.93589 (6)	0.02028 (17)	
N5	0.02224 (6)	0.36682 (9)	0.88352 (6)	0.01523 (17)	
H5	0.0796 (9)	0.4033 (13)	0.8674 (9)	0.018*	
C21	0.31624 (7)	0.23319 (10)	0.88312 (7)	0.01323 (18)	
C22	0.37511 (8)	0.36134 (11)	0.89410 (7)	0.0173 (2)	
H22	0.3397	0.4520	0.8913	0.021*	
C23	0.48377 (8)	0.35967 (12)	0.90890 (8)	0.0207 (2)	
H23	0.5223	0.4479	0.9161	0.025*	
C24	0.53540 (8)	0.22716 (12)	0.91305 (8)	0.0202 (2)	
H24	0.6099	0.2239	0.9260	0.024*	
C25	0.47790 (7)	0.09954 (11)	0.89823 (7)	0.0172 (2)	
H25	0.5143	0.0098	0.8995	0.021*	
C26	0.36771 (7)	0.09835 (10)	0.88136 (7)	0.01375 (18)	
C31	0.21410 (7)	−0.07550 (10)	0.88061 (7)	0.01417 (18)	
C32	0.16781 (8)	−0.21063 (11)	0.85663 (8)	0.0187 (2)	
H32	0.1011	−0.2310	0.8730	0.022*	
C33	0.21824 (9)	−0.31577 (11)	0.80897 (8)	0.0219 (2)	
H33	0.1863	−0.4078	0.7930	0.026*	
C34	0.31554 (9)	−0.28566 (11)	0.78484 (8)	0.0218 (2)	
H34	0.3509	−0.3571	0.7527	0.026*	
C35	0.36072 (8)	−0.15065 (11)	0.80807 (8)	0.0181 (2)	
H35	0.4267	−0.1305	0.7900	0.022*	
C36	0.31255 (7)	−0.04244 (10)	0.85732 (7)	0.01404 (18)	
N51	−0.04575 (6)	0.59431 (9)	0.85591 (6)	0.01663 (18)	
C52	−0.12294 (8)	0.69109 (11)	0.85240 (7)	0.01701 (19)	
H52	−0.1109	0.7889	0.8352	0.020*	
C53	−0.21986 (8)	0.65325 (11)	0.87304 (7)	0.01609 (19)	
C54	−0.23889 (8)	0.51174 (11)	0.90003 (8)	0.0181 (2)	
H54	−0.3054	0.4849	0.9143	0.022*	
C55	−0.15953 (7)	0.41067 (11)	0.90574 (8)	0.01687 (19)	
H55	−0.1691	0.3131	0.9251	0.020*	
C56	−0.06433 (7)	0.45741 (10)	0.88190 (7)	0.01390 (18)	
N57	−0.30275 (7)	0.76288 (10)	0.86450 (7)	0.02042 (19)	
O58	−0.39181 (6)	0.72147 (10)	0.86834 (7)	0.0296 (2)	
O59	−0.27849 (7)	0.89005 (9)	0.85351 (7)	0.02897 (19)	

### Atomic displacement parameters (Å^2^)

**Table:**

	*U*^11^	*U*^22^	*U*^33^	*U*^12^	*U*^13^	*U*^23^
N2	0.0173 (4)	0.0141 (4)	0.0190 (4)	0.0005 (3)	0.0079 (3)	0.0029 (3)
C3	0.0147 (4)	0.0141 (4)	0.0099 (4)	0.0007 (3)	0.0014 (3)	0.0000 (3)
N3	0.0138 (4)	0.0149 (4)	0.0119 (4)	−0.0002 (3)	0.0020 (3)	−0.0001 (3)
C4	0.0150 (4)	0.0153 (4)	0.0119 (4)	0.0004 (3)	0.0014 (3)	0.0005 (3)
O4	0.0173 (3)	0.0184 (3)	0.0263 (4)	−0.0001 (3)	0.0073 (3)	0.0053 (3)
N5	0.0124 (4)	0.0150 (4)	0.0187 (4)	−0.0001 (3)	0.0039 (3)	0.0016 (3)
C21	0.0137 (4)	0.0162 (4)	0.0096 (4)	−0.0006 (3)	0.0015 (3)	0.0010 (3)
C22	0.0191 (5)	0.0161 (4)	0.0173 (5)	−0.0019 (3)	0.0049 (4)	−0.0011 (4)
C23	0.0189 (5)	0.0223 (5)	0.0217 (5)	−0.0066 (4)	0.0055 (4)	−0.0032 (4)
C24	0.0138 (4)	0.0281 (5)	0.0186 (5)	−0.0021 (4)	0.0024 (4)	0.0007 (4)
C25	0.0154 (4)	0.0212 (5)	0.0152 (4)	0.0021 (4)	0.0030 (3)	0.0035 (4)
C26	0.0156 (4)	0.0159 (4)	0.0097 (4)	0.0004 (3)	0.0021 (3)	0.0023 (3)
C31	0.0161 (4)	0.0135 (4)	0.0130 (4)	0.0018 (3)	0.0028 (3)	0.0028 (3)
C32	0.0199 (5)	0.0153 (4)	0.0211 (5)	−0.0014 (4)	0.0041 (4)	0.0028 (4)
C33	0.0280 (5)	0.0139 (4)	0.0239 (5)	−0.0009 (4)	0.0054 (4)	0.0021 (4)
C34	0.0294 (5)	0.0156 (5)	0.0222 (5)	0.0044 (4)	0.0093 (4)	0.0017 (4)
C35	0.0197 (4)	0.0170 (4)	0.0184 (5)	0.0039 (4)	0.0057 (4)	0.0035 (4)
C36	0.0155 (4)	0.0134 (4)	0.0128 (4)	0.0015 (3)	0.0014 (3)	0.0037 (3)
N51	0.0172 (4)	0.0150 (4)	0.0171 (4)	0.0001 (3)	0.0017 (3)	0.0015 (3)
C52	0.0201 (4)	0.0158 (4)	0.0139 (4)	0.0010 (4)	−0.0001 (3)	0.0002 (3)
C53	0.0168 (4)	0.0192 (5)	0.0109 (4)	0.0048 (3)	−0.0012 (3)	−0.0018 (3)
C54	0.0146 (4)	0.0223 (5)	0.0173 (5)	0.0006 (4)	0.0024 (3)	−0.0004 (4)
C55	0.0151 (4)	0.0174 (4)	0.0180 (5)	−0.0011 (3)	0.0028 (4)	0.0010 (4)
C56	0.0142 (4)	0.0148 (4)	0.0118 (4)	0.0006 (3)	0.0001 (3)	0.0000 (3)
N57	0.0221 (4)	0.0247 (4)	0.0131 (4)	0.0081 (3)	−0.0003 (3)	−0.0017 (3)
O58	0.0187 (4)	0.0389 (5)	0.0316 (5)	0.0099 (3)	0.0057 (3)	0.0046 (4)
O59	0.0345 (5)	0.0193 (4)	0.0306 (5)	0.0084 (3)	−0.0002 (3)	−0.0015 (3)

### Geometric parameters (Å, °)

**Table:**

N2—C3	1.3591 (12)	C31—C36	1.4031 (13)
N2—C31	1.4213 (12)	C32—C33	1.3891 (14)
N2—H2	0.892 (11)	C32—H32	0.9500
C3—N3	1.2858 (12)	C33—C34	1.3878 (15)
C3—C4	1.5276 (13)	C33—H33	0.9500
N3—C21	1.4186 (12)	C34—C35	1.3853 (15)
C4—O4	1.2211 (11)	C34—H34	0.9500
C4—N5	1.3575 (12)	C35—C36	1.4048 (13)
N5—C56	1.3958 (12)	C35—H35	0.9500
N5—H5	0.879 (11)	N51—C52	1.3348 (13)
C21—C22	1.3987 (13)	N51—C56	1.3419 (12)
C21—C26	1.4122 (13)	C52—C53	1.3812 (14)
C22—C23	1.3864 (14)	C52—H52	0.9500
C22—H22	0.9500	C53—C54	1.3870 (14)
C23—C24	1.3883 (15)	C53—N57	1.4639 (13)
C23—H23	0.9500	C54—C55	1.3794 (13)
C24—C25	1.3864 (14)	C54—H54	0.9500
C24—H24	0.9500	C55—C56	1.3996 (13)
C25—C26	1.4050 (13)	C55—H55	0.9500
C25—H25	0.9500	N57—O58	1.2265 (12)
C26—C36	1.4872 (13)	N57—O59	1.2288 (13)
C31—C32	1.3933 (13)		
			
C3—N2—C31	123.51 (8)	C33—C32—C31	120.63 (9)
C3—N2—H2	112.5 (8)	C33—C32—H32	119.7
C31—N2—H2	116.2 (8)	C31—C32—H32	119.7
N3—C3—N2	130.55 (9)	C34—C33—C32	119.62 (10)
N3—C3—C4	116.30 (8)	C34—C33—H33	120.2
N2—C3—C4	113.09 (8)	C32—C33—H33	120.2
C3—N3—C21	124.20 (8)	C35—C34—C33	119.47 (9)
O4—C4—N5	126.12 (9)	C35—C34—H34	120.3
O4—C4—C3	121.37 (8)	C33—C34—H34	120.3
N5—C4—C3	112.50 (8)	C34—C35—C36	122.42 (9)
C4—N5—C56	129.34 (8)	C34—C35—H35	118.8
C4—N5—H5	111.8 (8)	C36—C35—H35	118.8
C56—N5—H5	118.9 (8)	C31—C36—C35	116.95 (9)
C22—C21—C26	119.56 (8)	C31—C36—C26	124.20 (8)
C22—C21—N3	112.79 (8)	C35—C36—C26	118.85 (8)
C26—C21—N3	127.65 (8)	C52—N51—C56	117.84 (8)
C23—C22—C21	121.75 (9)	N51—C52—C53	121.94 (9)
C23—C22—H22	119.1	N51—C52—H52	119.0
C21—C22—H22	119.1	C53—C52—H52	119.0
C22—C23—C24	119.05 (9)	C52—C53—C54	120.14 (9)
C22—C23—H23	120.5	C52—C53—N57	119.53 (9)
C24—C23—H23	120.5	C54—C53—N57	120.33 (9)
C25—C24—C23	119.80 (9)	C55—C54—C53	118.79 (9)
C25—C24—H24	120.1	C55—C54—H54	120.6
C23—C24—H24	120.1	C53—C54—H54	120.6
C24—C25—C26	122.26 (9)	C54—C55—C56	117.43 (9)
C24—C25—H25	118.9	C54—C55—H55	121.3
C26—C25—H25	118.9	C56—C55—H55	121.3
C25—C26—C21	117.41 (9)	N51—C56—N5	112.52 (8)
C25—C26—C36	118.41 (8)	N51—C56—C55	123.85 (9)
C21—C26—C36	124.09 (8)	N5—C56—C55	123.63 (9)
C32—C31—C36	120.89 (9)	O58—N57—O59	124.44 (9)
C32—C31—N2	116.34 (8)	O58—N57—C53	117.77 (9)
C36—C31—N2	122.77 (9)	O59—N57—C53	117.79 (9)
			
C31—N2—C3—N3	−40.22 (16)	C32—C33—C34—C35	−0.43 (16)
C31—N2—C3—C4	142.83 (9)	C33—C34—C35—C36	1.23 (16)
N2—C3—N3—C21	−9.51 (16)	C32—C31—C36—C35	0.77 (14)
C4—C3—N3—C21	167.36 (8)	N2—C31—C36—C35	−179.14 (9)
N3—C3—C4—O4	−177.99 (9)	C32—C31—C36—C26	−179.29 (9)
N2—C3—C4—O4	−0.58 (13)	N2—C31—C36—C26	0.80 (14)
N3—C3—C4—N5	1.05 (12)	C34—C35—C36—C31	−1.38 (14)
N2—C3—C4—N5	178.47 (8)	C34—C35—C36—C26	178.68 (9)
O4—C4—N5—C56	−0.22 (17)	C25—C26—C36—C31	151.43 (9)
C3—C4—N5—C56	−179.21 (9)	C21—C26—C36—C31	−32.08 (14)
C3—N3—C21—C22	−153.01 (9)	C25—C26—C36—C35	−28.63 (13)
C3—N3—C21—C26	26.82 (15)	C21—C26—C36—C35	147.86 (9)
C26—C21—C22—C23	−3.56 (15)	C56—N51—C52—C53	−0.98 (14)
N3—C21—C22—C23	176.28 (9)	N51—C52—C53—C54	1.09 (15)
C21—C22—C23—C24	−0.09 (15)	N51—C52—C53—N57	−177.84 (9)
C22—C23—C24—C25	2.66 (15)	C52—C53—C54—C55	0.01 (15)
C23—C24—C25—C26	−1.61 (15)	N57—C53—C54—C55	178.94 (9)
C24—C25—C26—C21	−1.98 (14)	C53—C54—C55—C56	−1.11 (14)
C24—C25—C26—C36	174.74 (9)	C52—N51—C56—N5	−179.63 (8)
C22—C21—C26—C25	4.48 (13)	C52—N51—C56—C55	−0.22 (15)
N3—C21—C26—C25	−175.34 (9)	C4—N5—C56—N51	177.97 (9)
C22—C21—C26—C36	−172.04 (9)	C4—N5—C56—C55	−1.44 (16)
N3—C21—C26—C36	8.14 (15)	C54—C55—C56—N51	1.27 (15)
C3—N2—C31—C32	−134.33 (10)	C54—C55—C56—N5	−179.39 (9)
C3—N2—C31—C36	45.58 (14)	C52—C53—N57—O58	169.30 (9)
C36—C31—C32—C33	−0.04 (15)	C54—C53—N57—O58	−9.64 (14)
N2—C31—C32—C33	179.87 (10)	C52—C53—N57—O59	−10.38 (14)
C31—C32—C33—C34	−0.14 (16)	C54—C53—N57—O59	170.68 (9)

### Hydrogen-bond geometry (Å, °)

**Table:**

*D*—H···*A*	*D*—H	H···*A*	*D*···*A*	*D*—H···*A*
N2—H2···O4	0.89 (1)	2.24 (1)	2.7041 (11)	112 (1)
N2—H2···O4^i^	0.89 (1)	2.26 (1)	3.0725 (11)	152 (1)
N5—H5···N3	0.88 (1)	2.11 (1)	2.6191 (11)	116 (1)
C55—H55···O4	0.95	2.33	2.9266 (12)	120
C32—H32···N51^ii^	0.95	2.47	3.3000 (13)	146

Symmetry codes: (i) −*x*, −*y*, −*z*+2; (ii) *x*, *y*−1, *z*.
